# Supplementary material for: Proteobacteria and Firmicutes Secreted Factors Exert Distinct Effects on Pseudomonas aeruginosa Infection under Normoxia or Mild Hypoxia
Source: Metabolites. 2022 May 17;12(5):449. doi: 10.3390/metabo12050449 (PMC9146490; doi:10.3390/metabo12050449)
Supplement: Supplementary file 1 [file metabolites-12-00449-s001.zip › metabolites-1690746-supplementary.pdf]

Supplementary Materials:

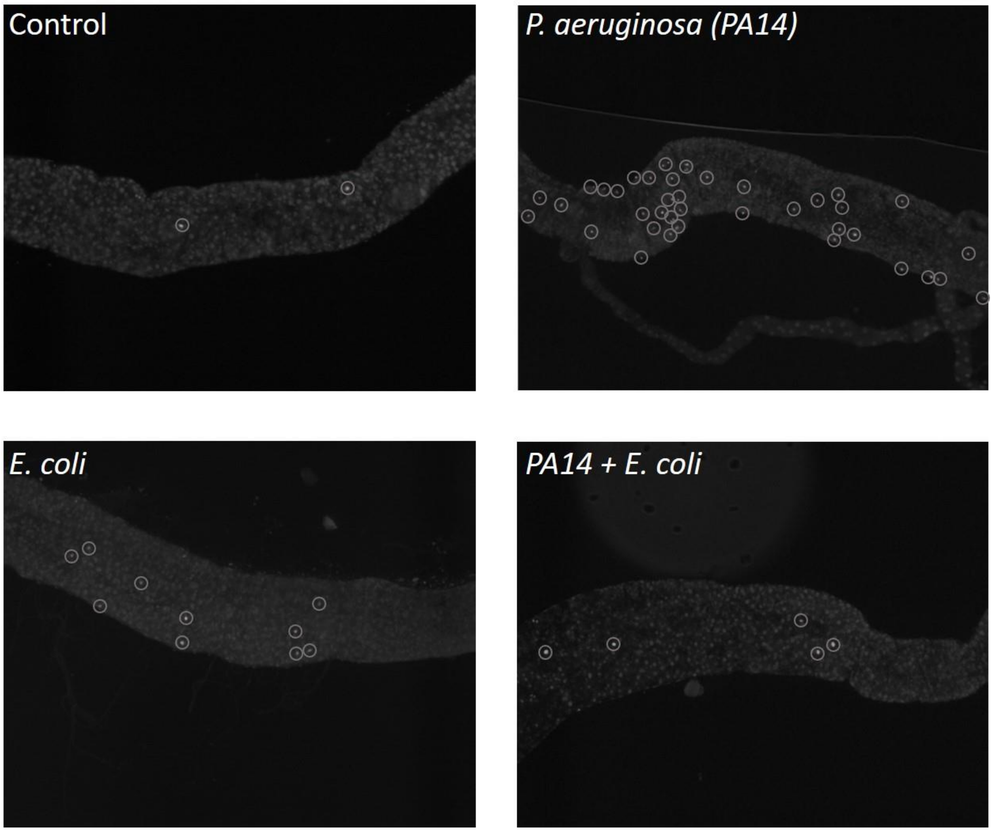

Supplementary Figure S1. *P. aeruginosa* (PA14) and *E. coli* (MGH) display antagonistic interactions in the fly midgut, leading to a reduction in the numbers of intestinal mitosis. Confocal images of mitotic cells in the fly midgut, immunostained using anti-pH3 antibody, 48h-post infection with either *P. aeruginosa* (PA14) only or *E. coli* (MGH) only or *P. aeruginosa* + *E. coli*, at 20X magnification. Grey outlines encircle all $pH3^+$ cells.

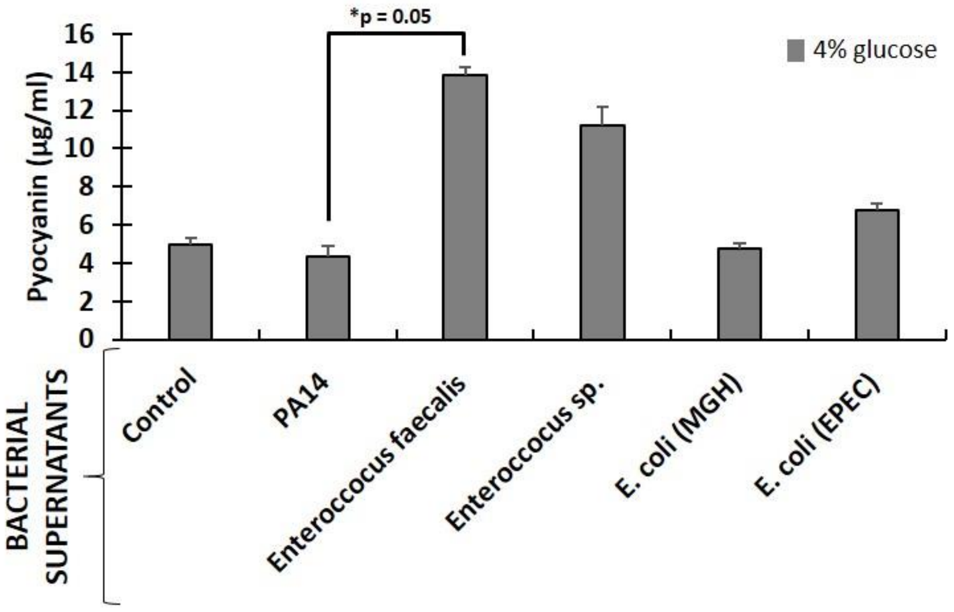

Supplementary Figure S2. Secreted factors from *Enterococcus faecalis* and *Enterococcus* sp., tentatively increase pyocyanin production by *P. aeruginosa* (PA14) in culture, in the presence of glucose. PA14-produced pyocyanin concentration in cultures of *P. aeruginosa* (PA14) in the presence of 4% glucose at 24 hours of PA14 alone (control) or in the presence of bacterial supernatants derived from cultures of either PA14, *Enterococcus faecalis, Enterococcus sp., E. coli* (MGH), *E. coli* (EPEC). Error bars represent standard deviation of the mean. Statistical analysis was performed using the one-way ANOVA test and Dunn's multiple comparisons test. Statistical significance indicated as *p=0.05.
